# Long Term Cognitive Function After Cardiac Arrest: A Mini-Review

**DOI:** 10.3389/fnagi.2022.885226

**Published:** 2022-05-26

**Authors:** Guri Hagberg, Håkon Ihle-Hansen, Else Charlotte Sandset, Dag Jacobsen, Henning Wimmer, Hege Ihle-Hansen

**Affiliations:** ^1^Department of Medical Research, Baerum Hospital Vestre Viken Hospital Trust, Drammen, Norway; ^2^Oslo Stroke Unit, Department of Neurology, Oslo University Hospital, Ullevål, Norway; ^3^Department of Medicine, Baerum Hospital Vestre Viken Hospital Trust, Drammen, Norway; ^4^Department of Acute Medicine, Oslo University Hospital, Ullevål, Norway

**Keywords:** cardiac arrest, cognition, dementia, hypoxic brain injury, cognitive impaiment

## Abstract

Out-of-hospital cardiac arrest (OHCA) is a leading cause of mortality worldwide. With better pre- and inhospital treatment, including cardiopulmonary resuscitation (CPR) as an integrated part of public education and more public-access defibrillators available, OHCA survival has increased over the last decade. There are concerns, after successful resuscitation, of cerebral hypoxia and degrees of potential acquired brain injury with resulting poor cognitive functioning. Cognitive function is not routinely assessed in OHCA survivors, and there is a lack of consensus on screening methods for cognitive changes. This narrative mini-review, explores available evidence on hypoxic brain injury and long-term cognitive function in cardiac arrest survivors and highlights remaining knowledge deficits.

## Introduction

Cardiac arrest, defined as sudden cessation of cardiac activity with loss of consciousness, breathing, and no signs of circulation, is a leading cause of mortality worldwide. The condition rapidly progresses to sudden death if untreated with immediate cardiopulmonary resuscitation (CPR) and defibrillation (if indicated) (Kuller, 1980). The exact global burden, meaning mortality and morbidity, of out-of-hospital cardiac arrest (OHCA) to public health is unclear, due to the variability of emergency medical services (EMS) presentations, and regional variations in both systems and survival (Myat et al., 2018; Dyson et al., 2019). The estimated incidence is 275 000 people in Europe and 356 000 in the United States, with approximately 10% surviving to hospital discharge. Incidence increases with age and more commonly occurs in men. The etiology is predominantly cardiovascular in nature, with ischemic heart disease accounting for 60–80% of cases (Benjamin et al., 2018).

Scandinavian registry data including data from 2001 to 2010, show an increased survival in patients with OHCA from 10 to 30%, especially in patients with diagnosis suitable for defibrillation, such as ventricular fibrillation and pulseless ventricular tachycardia. Improvement in survival rates is likely attributable to the application of widespread CPR training and public-access defibrillators (Wissenberg et al., 2013). Additionally, the adoption of standardized post-resuscitation care, including goal-directed therapy with therapeutic hypothermia and increasing access to percutaneous coronary intervention for OCHA presentations of acute coronary syndromes, has also been shown to improve overall survival (Lund-Kordahl et al., 2010). The Norwegian Cardiac Arrest Registry (NorCAR) was established in 2002 and received status as a mandatory national health registry in 2013 (Tjelmeland et al., 2020), as the world’s first mandatory population-based cardiac arrest registry. According to this register, of the 3,405 attempted resuscitations in 2018, 1018 were brought to the hospital, and 405 survived (12%) more than 30 days.

Hypoxic brain injury is the major cause of death and disability in admitted patients post-OHCA after successful resuscitation (Dragancea et al., 2013). To improve the neurological prognosis, guidelines recommend targeted temperature management (TTM) after return of spontaneous circulation (Nolan et al., 2021). The Cerebral performance category (CPC) (Mak et al., 2016), adapted from the Glasgow coma Scale, is the predominantly employed score to assess neurological outcomes in OHCA survivors. The score consists of a five-point scale describing different functional statuses, where a score of 1 or 2 is considered a good outcome, indicating independence in activities of daily living. In 2018, 80% of the survivors in NorCAR had a good neurological outcome (CPC score 1-2) (Tjelmeland et al., 2020). However, the CPC score does not assess cognitive function, a relevant domain form a patient’s perspective. In parallel, functional disability after stroke are evaluated with the modified Rankin scale (mRS) (Wilson et al., 2005), and in cases with excellent clinical recovery at 3 months (mRS = 0-1, no disability), the occurrence of cognitive impairment is prevalent (Jokinen et al., 2015). Eemphasizing the importance of long-term outcomes after OHCA, there is a need for good prediction models including cognition.

The majority of patient prognostication takes place in intensive care units, especially regarding the decision to withdraw from life-sustaining treatment in unconscious patients. Prognostication strategy algorithms exist; however, their utility in predicting poor outcome in patients suffering OHCA is uncertain (Cronberg et al., 2020; Nakstad et al., 2020). In the Norwegian Cardio-Respiratory Arrest Study (NORCAST) study, 54% out of the 259 comatose patients survived to discharge. Only 3 (absence of pupillary reflexex, bilateral absent N20 somato-sensory evoked potentials and increased neuron-specific enolase later than 24 h to >80 μg/L) out of 15 clinical, neurological, and biochemical predictors predicted poor outcomes with no false-positive rates (Nakstad et al., 2020).

The brain is vulnerable to hypoxic injury, and neuronal cell areas are more susceptible (Cronberg et al., 2020). The total burden of brain lesions after OHCA is unclear, largely due to the technical challenges in performing diagnostic neuroradiology in critically ill patients and hence, these are often limited to patients without neurological recovery after sedation. In small imaging and autopsy studies, the severity of findings is highly individual and depends on several factors, including the time to reperfusion and imaging. Magnetic resonance imaging (MRI) assessment on recovery has historically been limited due to pacemakers as a contraindication to an MRI scan (Muttikkal and Wintermark, 2013). Most registries, including NorCAR, do not routinely register neuroimaging findings or standardized cognitive assessments. Recent guidelines suggest using brain imaging for prognostication only in centers where specific experience is available (Nolan et al., 2021).

As survival rates from OHCA improve, there are increasing concerns regarding the impact on cognitive function following successful cardiopulmonary resuscitation and more data are needed. To “save the heart but lose the brain,” a patient’s ability to learn, think and reflect, can have significant consequences longer term. We aim to explore the currently available evidence on the long-term cognitive function and hypoxic brain injury in survivors after cardiac arrest and further identify the remaining knowledge gaps. This narrative mini review will focus on OHCA and long-term cognitive outcomes defined as longer than three months post-arrest.

## Cognitive Function After Cardiac Arrest

**FIGURE 1:**
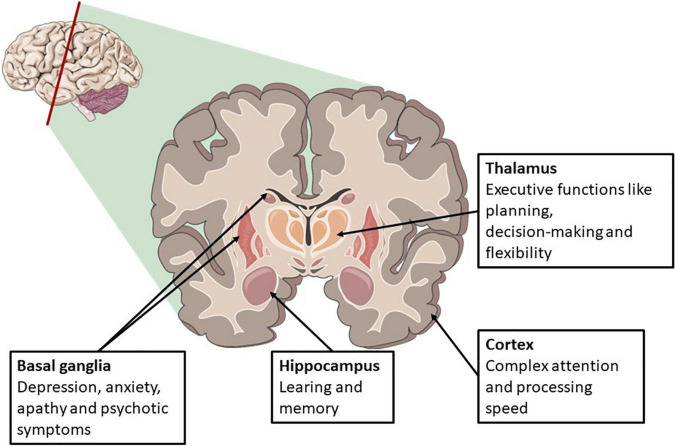
Areas susceptible for global anoxic-ischemic brain injury and related cognitive symptoms. Illustration adapted from servier medial art.

Global or domain-specific cognitive test can be used when assessing cognition. There are numerous tests and test batteries, some generic other disease-specific. The most sensitive areas to hypoxic injury are the cortex and basal ganglia, followed by the hippocampus, thalamus, and brainstem. These areas are linked to cognitive domains, shown in Figure 1. Cognitive functions are dependent on complex interactions between cortical and subcortical sites across different brain networks. These networks are widely distributed across the brain, frequently intersecting, and overlapping, so that one lesion could affect multiple networks (Nakstad et al., 2020). By that, a global screening tool, including most cognitive domains, might be feasible.

Current evidence available on cognitive function after cardiac arrest is summarized in Table 1.

**TABLE 1:** Excerpts from relevant studies according to level of evidence.

Systematic Reviews			

References	Population size	Measurement and outcome	Conclusion/interpretation
Resuscitation (Moulaert et al., 2009)	3 studies n = 45, 57 and 58	Different battery of neuropsychological tests. Cognitive problems were found in 42, 48, and 50% of participants.	There are few good studies. Cognitive problems, in particular memory problems, seem common in survivors of out-of-hospital cardiac arrest.

**Randomized control trials and their substudies**			

Circulation (Lilja et al., 2015)	n = 652 The study also included an age-matched control group, with known cardiovascular disease. 50% were alive at follow-up, 90% attended in the structured examination at 6 months.	6 months—follow-up, domain-specific cognitive tests. 50% of the OHCA obtained a normal score at the memory assessment. Both OHCA groups performed worse than the control group on test for attention and mental speed.	No gold standard tests, or combination of tests currently exists. Cognitive function was comparable in the two temperature management groups. Cognitive impairment detected in cardiac arrest survivors was also common in matched control subjects with known cardiovascular disease.
JAMA (Cronberg et al., 2015)	*n* = 950 50% were alive at follow-up, 90% attended in the structured examination at 6 months.	6 months—follow-up. MMSE, IQCODE. Mean MMSE score was 28, which is considered normal compared to an age matched control group. Relatives report a minor reduction from previous level in cognition using the IQCODE.	Need of tests and scales that can improve the discrimination of the degree of neurologic recovery. Cognitive function was similar in both intervention groups, but many patients and observers reported impairment not detected previously by standard outcome scales.
Resuscitation (Evald et al., 2019)	*n* = 79	6 months follow-up. Rey Auditory-Verbal Learning Test (RAVLT) and Rey–Osterreith Complex Figure Test (ROCFT) for learning and memory; WAISIV Digit Span17 and Trail Making Test A & B (TMT-A & B) for attention; and D-KEFS Verbal Fluency for executive functions. TTM48 was associated with a significant better performance on three of 13 cognitive tests specific to memory, namely the RAVALT and immediate and delayed ROCFT tests.	TTM48 was associated with reduced memory retrieval deficits and lower relative risk of cognitive impairment six months after OHCA compared to standard TTM24.

**Cohort Studies**			

Resuscitation (Caro-Codón et al., 2018)	n = 79	1-year follow-up. MoCA, TMT-B, modified IQCODE, Zarit Caregigiven Burden interview, CPC, mRS. 54.4% scored below the usual cut-off for the diagnosis of MCI.	There is a high prevalence of long-term cognitive deficits and functional limitations in OHCA survivors. CPC or mRS, are crude and lack sensitivity to detect most of these deficits.
Resuscitation (Ørbo et al., 2014)	n = 30	3 months and 1 year follow-up. Neuropsychological tests for memory, executive function, and psychomotor speed. Memory impairments were the most common symptom, and stable from 3 to 12 months.	While systematic, early screening of cognitive performance has been recommended in recent p ost-resuscitation guideline, these concepts are not implemented in most places.
Resuscitation (Buanes et al., 2015)	n = 33	4-year follow-up. Cambridge Neuropsychological Test Automated Battery. 25% had cognitive impairment. Short-term memory was predominantly affected.	Cognitive impairment four years after cardiac arrest affected more than one-quarter of the patients.
Resusitation (Byron-Alhassan et al., 2021)	n = 79	Neuropsychological Assessment Battery (NAB). 43% s were Cognitively impaired (in the lowest decile on a global measure of cognitive functioning). Attention, memory, language and executive function were affected.	OHCA survivors - even those with seemingly good neurological recovery are at risk for cognitive impairment. Cognitive rehabilitation may be an important consideration post-OHCA.
Chest (Peskine et al., 2021)	n = 139	3-, 6-, 12- and 18-month follow-up. MMSE, Repeatable Battery for the assessment of Neuropsychological Status (RBANS) and the Frontal Assessment Battery. At 18 moths 20% had cognitive disabilities (MMSE < 25).	OCHA have good long-term prognosis, some patients improved until 18 moths post OHCA. Whether specific rehabilitation programs for these patients could improve outcome remains to be determined.

*MMSE, Mini-Mental State Exam; MoCA, Montreal Cognitive Assessment; TMT-B, Trail-making test-B; IQCODE, Informant Questionnaire on Cognitive Decline in the Elderly; CPC, Cerebral Performance Category; mRS, modified Rankin Scale.*

We identified only one systematic review from 2009 based on 28 papers from 1980 to 2006, describing the current evidence on the measured frequency and nature of cognitive impairments in OHCA survivors. Both design, participant, quality, and cognitive measures varied considerably in the studies included in the review. Only three studies with a small sample size (range 45–58) assessed cognitive function using a neuropsychological test battery. Cognitive problems were common and present in 42–50% of the participants (Moulaert et al., 2009).

Two substudies (Cronberg et al., 2015; Lilja et al., 2015) based on a large randomized controlled trial (RCT) (Nielsen et al., 2013), with prespecified secondary outcomes on cognition, randomized OHCA survivors to different temperature regimes (33 vs. 36°C), during the first 36 hours, with six months follow-up. Cognitive function did not differ in the two temperature management groups. In the first study, both OHCA groups performed worse than the age-matched control group, with known cardiovascular disease on tests for attention and mental speed (Lilja et al., 2015). In the other study (Cronberg et al., 2015), relatives reported a minor reduction from previous level in cognition using a modified version of the Informant Questionnaire on Cognitive Decline in the Elderly (IQCODE) (Jorm et al., 1991).

In another RCT comparing 48 h of hypothermia with 24 h of hypothermia in patients post cardiac arrest, the subgroup of patients with CPC score ≤2 at 6 months, demonstrated a longer duration of hypothermia (48 h) was associated with a lower risk of cognitive impairment (Evald et al., 2019).

Five small cohort studies with 79, 30, 33, 79, and 139 participants respectively, ranging from one to four years of follow-up, and using different global cognitive screening tools, reported some degree of cognitive impairment in 25–55% of the OHCA survivors (Ørbo et al., 2014; Buanes et al., 2015; Caro-Codón et al., 2018; Byron-Alhassan et al., 2021; Peskine et al., 2021). Caro-Codón et al. (2018) used a set of outcome measures often used in dementia diagnostic workup and compared their findings with the functional score CPC and mRS. Half of the patients scored below the usual limit for the diagnosis of mild cognitive impairment (MCI) and mRS did not detect these impairments (Caro-Codón et al., 2018). Ørbo et al. (2014) identified memory impairments as the most common symptom, and the impairments were stable from three to 12 months. Buanes et al. (2015) reported that more than one-quarter of the patients had cognitive impairment with short-term memory predominantly affected during a four-year follow-up. Byron-Alhassan et al. (2021) compared OHCA survivors to patients who had experienced a myocardial infarction and found a six times higher rate of cognitive impairment in the OHCA group. Peskine et al. (2021) report that 20% of OHCA survivors (with GCS > 12 in the 2 weeks post arrest) had cognitive impairment at 18 months, but observed in general improvement from 3 to 18 months.

Most studies highlight the lack of guideline recommendations on how to perform cognitive screening post-resuscitation.

## Brain Pathology on Imaging After Cardiac Arrest

Neuroradiology is so far not an established part of the diagnostic assessment after cardiac arrest. Different patterns of injury have been reported in advanced imaging, depending on modality and timing of the assessment. Computed Tomography (CT) scan showing signs of edema; diffuse graying of the cerebral hemispheres, and loss of gray-white matter differentiation, are known predictors of poor outcomes after cardiac arrest (Nolan et al., 2021). Isolated cerebral edema, however, may not be a bad prognostic sign even if accompanied by late status epilepticus (Sunde et al., 2006). Ischemic lesions in the border zones between two major arterial territories are usually associated with hypoperfusion and described as watershed infarction on MR and CT. Diffuse hypoxic-ischemic changes involving gray matter in both cerebral hemispheres are a frequent finding after cardiac arrest of unknown duration (Keijzer et al., 2018).

A small case-control study (n = 12) on cardiac arrest survivors showed an extensive reduction of gray matter volumes on MRI compared to age- and sex-matched controls (Horstmann et al., 2010). A retrospective study including 50 cases with cardiac arrest, reviewed imaging findings of MRI reports concluding hypoxic-ischemic brain injury, and identified diffuse cortical and deep gray matter pattern of injury as the most common radiologic finding in those with poor outcomes. Lesions in the cerebellum and brainstem were seen in 30 and 7% of cases, respectively. In general, most patients had a poor clinical outcome (mRS 4-6) regardless of the observed pattern of injury, however a basal ganglia pattern without cortical involvement and watershed pattern could be an exception (Muttikkal and Wintermark, 2013).

A review published in Resuscitation 2018 aiming to value CT, MRI, and Positron Emission Tomography (PET) as an early prediction method of neurological outcome of comatose cardiac arrest survivors, identified 51 articles, 21 using CT, 27 MRI, one with both CT and MRI and two with PET imaging. CT or MRI with diffusion weighted imaging (DWI) within 1–3 days of cardiac arrest, demonstrating involvement of more than 10% of the brain with cytotoxic edema, may offer early prediction for adverse outcomes (Keijzer et al., 2018). Similarly, a more recent meta-analysis (Lopez Soto et al., 2020) and retrospective single center observational study (Schick et al., 2022) have demonstrated the utility of both CT findings of loss of gray-white matter differentiation and MRI with DWI and fluid attenuated inversion recovery (FLAIR) sequencing in neuro-prognostication post-cardiac arrest. No long-term prediction data are included in either study.

Small cohort studies have examined the association between brain atrophy and cognition in OHCA survivors. The hippocampus and cortical volume were smaller in OHCA survivors than in healthy controls at three months, corresponding to observed cognitive impairments, mostly memory deficits. They conclude that neuroimaging studies of long-term OHCA survivors are warranted to guide the development of diagnostic and treatment options (Ørbo et al., 2018, 2019).

## Discussion

Up to 50% of OHCA survivors have cognitive impairments, often mild, but largely undetected by contemporary functional outcome measurements, notably the Cerebral Performance Category. Understanding of neuroradiologic findings after cardiac arrest and their relationship to longer term neurological outcomes still in its infancy. Observed patterns of injury, such as diffuse cortical and deep gray matter injury are noted and may related to later clinical findings in cognitive domains involved in executive functions, memory, and processing speed.

Cardiac arrest trials have traditionally reported outcomes that focus on survival and crude functional impairments. In addition, there is lack of consistency in outcome reporting. Recommended primary outcomes for resuscitation science studies, published in a consensus statement from the American Heart Association (AHA) 2011 (Becker et al., 2011), include global and domain-specific cognitive tests. Mini-mental state examination (MMSE) (Folstein et al., 1975) at discharge and follow-up is recommended as standard in clinical practice. MMSE is a global screening tool and has shown limited value in mild cognitive impairment (MCI) and does not assess executive function or complex attention, including processing speed and may not be the test of choice in patients with hypoxic brain injury (Dong et al., 2010). Further, AHA recommends TMT-A and B (Rm, 1958), and specific testing for memory (Rey Auditory Verbal Learning Test (RAVLT)(Ryan and Geisser, 1986) and attention (Digit Symbol Substitution Test (DSST) (Bettcher et al., 2011). The European Resuscitation Council and the European Society of Intensive Care Medicine have collaborated to produce post-resuscitation care guidelines (Nolan et al., 2021). The specific recommendations are screening for cognitive impairments using the Montreal Cognitive Assessment (MoCA) (Nasreddine et al., 2005) test and screening for emotional problems using the Hospital Anxiety and Depression Scale (HADS) (Snaith and Zigmond, 1986). Referral to a neuropsychological assessment or psychologist or psychiatrist if necessary are also recommended. The guidelines reflect the heterogeneity of the evidence.

Diagnostic criteria can be used to define the severity of cognitive symptoms and identify patients in need of cognitive rehabilitation. Some general, other disease-specific diagnostic criteria exist, including biomarkers such as MRI to include proposed etiology. As no disease-specific criteria exist after cardiac arrest, general criteria could be used, and the Diagnostic and Statistical Manual of Mental Disorders, DSM-5, is often used in clinical settings (Sachdev et al., 2014). No study identified used diagnostic criteria, making comparison difficult and prevalence estimates uncertain. According to DSM-5, there must be evidence of modest cognitive decline from a previous level in one or more domains, preferably documented by standardized neuropsychological testing. The distinction between mild and major neurocognitive disorder is the interference with independence in everyday activities. No specific tests are recommended in the DSM criteria, but test performance in mild neurocognitive disorder should fall in the range of 1–2 SD below the normative mean and below 2 SD for major. The symptoms must also be present for longer than six months.

In general, there is no linear relationship between changes on imaging and cognitive function. However, chronic changes like periventricular white matter changes, caused by small vessel disease, and atrophy or neurodegeneration, are associated with cognitive decline (Barber et al., 1999; Wardlaw et al., 2013). Cognitive decline and dementia are also common after stroke. A proposed model of mechanisms in post-stroke dementia includes the severity of the vascular insult itself and the patient’s total burden of brain pathology and cognitive reserve, together called resilience, prior to the insult. A patient’s cognitive reserve is highly dependent on age, education, and lifestyle factors. The total burden of pathological brain changes includes chronic vascular changes, atrophy, and prior stroke. A patient with high brain resilience, suffering stroke will only result in a diagnosis of dementia if the infarct is strategic (Mok et al., 2017). A similar model could be applied to cardiac arrest survivors; patients with cardiac arrest and high brain resilience will probably only develop dementia if global ischemia is severe. The median age in OHCA is 65 years, with ischemic heart disease accounting for 60–80%, implying a high vascular risk factor burden and chronic brain changes are also likely to be prevalent in a cardiac arrest population (Myat et al., 2018). As seen in the RCT from Lilja et al. (2015), cognitive impairment is as prevalent in an age-matched control group as in cardiac arrest survivors, but the OHCA survivors do worse on specific tests for attention and mental speed. This is in line with our knowledge that the most common pattern on MRI after hypoxic-ischemic include diffuse cortical and deep gray matter lesions, areas linked to different cognitive domains like executive functions and attention and processing speed (Figure 1).

Long-term data on cognition is needed to make good prediction models, and incorporate pre-arrest factors likely to influence cognition (such as cognitive impairments, genetics, education, comorbidities, or prior brain pathology). Imaging and cognitive assessment data are scarce and not included in national registries, and currently, data beyond six months is limited to small cohort studies.

## Future Perspectives

National Cardiac Arrest Registries need to include cognitive assessments and long-term follow-up. The cognitive test battery needs a domain-specific test, including attention, processing speed, learning, and memory. MoCA is a brief screening tool covering all these domains, and by adding a well-known screening tool for anxiety and depression, the most likely cognitive impairments after cardiac arrest will be covered. This screening could be done during the initial stay if possible, and if deficits are revealed repeated in a follow-up visit. In younger patients planning to return to work, more extensive neuropsychological testing might be necessary even if the initial screening is normal. Neuroimaging is a promising marker for long-term cognitive prognostics and should be a part of a throughout evaluation. Several ongoing trials with cognitive measurements, neuroimaging, and planned at least 3 months follow-up are registered at clinicaltrials.gov (Table 2). A dedicated multidisciplinary team offering OHCA survivors and their caregivers systematic psychological, cognitive, and specialized medical support for the first six months has shown promising results (Ørbo et al., 2018). Including follow-up of patients in National Cardiac Arrest Registry, will identify the actual burden of long-term cognitive deficits and subsequently identify patients who may benefit from long-term cognitive rehabilitation.

**TABLE 2:** Ongoing trials registered at clinicaltrials.gov.

Study and design NCT number	Planned population size	Primary aim	Cognitive tests	Neuroimaging	Follow-up
Brain Function After Cardiac Arrest (Measured With FMRI and Cognitive Tests) BRAINnHEART, cohort study, NCT03579498	60	Whether cognitive function is affected after cardiac arrest and whether it changes over time	CANTAB MoCa	functional MRI (fMRI)	12 months
Cracking Coma, cohort study, NCT03308305	100	To estimate the additional value of early MRI monitoring for the prediction of neurological outcome of comatose patients after cardiac arrest	Cognitive functioning as defined by professional Neuropsychological examination at 12 months	MRI of the brain at day 3, 7, and three months after cardiac arrest	12 months
The MOCHA Study: Multimodal Outcome CHAracterization in Comatose Cardiac Arrest Patients Data Registry and Tissue Repository, NCT03261089	2500	Develop an accurate and reliable assessment algorithm for determining neurologic prognosis in patients initially unconscious post-cardiac arrest, using multiple prognostic modalities at standardized time points	Cerebral Performance Category- Extended (CPC-E) MoCA	Neuroimaging at standardized time points – not specified	5 years
Influence of Cooling Duration on Efficacy in Cardiac Arrest Patients (ICECAP), RCT, NCT04217551	1800	Determine if increasing durations of induced hypothermia are associated with an increasing rate of good neurological outcomes	NIH Toolbox Crystallized Cognition Composite NIH Toolbox Fluid Cognition Composite Score processing in novel situations	unknown	90 d

## Conclusion

Cognitive impairments after OHCA are common and affect up to 50%. CPC is crude and lacks sensitivity to detect most of these deficits. As diffuse cortical and deep gray matter lesions were the most common findings on neuroimaging, cognitive domains involved in executive functions, memory, and processing speed needs to be addressed. More long-term data is required to develop good prognostic models, which could be in cohort studies or the registries. As of today, no standardized follow-up exists for the OHCA survivors, but recent guidelines recommends both cognitive screening and follow-up.

## Author Contributions

All authors listed have made a substantial, direct, and intellectual contribution to the work, and approved it for publication.

## Conflict of Interest

The authors declare that the research was conducted in the absence of any commercial or financial relationships that could be construed as a potential conflict of interest.

## Publisher’s Note

All claims expressed in this article are solely those of the authors and do not necessarily represent those of their affiliated organizations, or those of the publisher, the editors and the reviewers. Any product that may be evaluated in this article, or claim that may be made by its manufacturer, is not guaranteed or endorsed by the publisher.
